# External validation and comparison of Fetal Medicine Foundation competing‐risks model for small‐for‐gestational‐age neonate in the first trimester: multicenter cohort study

**DOI:** 10.1002/uog.29219

**Published:** 2025-04-14

**Authors:** P. Chaveeva, I. Papastefanou, T. Dagklis, N. Valiño, R. Revello, B. Adiego, J. L. Delgado, V. Kalev, I. Tsakiridis, C. Triano, M. Pertegal, A. Siargkas, B. Santacruz, C. de Paco Matallana, M. M. Gil

**Affiliations:** ^1^ Fetal Medicine Unit, Shterev Hospital Sofia Bulgaria; ^2^ Medical University Pleven Bulgaria; ^3^ Department of Women and Children's Health, Faculty of Life Sciences and Medicine King's College London London UK; ^4^ Third Department of Obstetrics and Gynecology, School of Medicine, Faculty of Health Sciences Aristotle University of Thessaloniki Thessaloniki Greece; ^5^ Department of Obstetrics and Gynecology Complejo Hospitalario Universitario A Coruña A Coruña Galicia Spain; ^6^ Department of Obstetrics and Gynecology Hospital Universitario Quirón, Pozuelo de Alarcón Madrid Spain; ^7^ Department of Obstetrics and Gynecology Hospital Universitario Fundación de Alcorcón, Alcorcón Madrid Spain; ^8^ Department of Obstetrics and Gynecology, Hospital Clínico Universitario ‘Virgen de la Arrixaca’, El Palmar Murcia Spain; ^9^ Faculty of Medicine Universidad de Murcia Murcia Spain; ^10^ Department of Obstetrics and Gynecology Hospital Universitario de Torrejón, Torrejón de Ardoz Madrid Spain; ^11^ Faculty of Medicine Universidad Francisco de Vitoria, Pozuelo de Alarcón Madrid Spain; ^12^ Department of Obstetrics and Gynecology Hospital Universitario La Paz Madrid Spain

**Keywords:** algorithm, Bayes' theorem, biomarker, fetal growth restriction, Fetal Medicine Foundation, FMF, prediction model, SGA, small‐for‐gestational age, survival model

## Abstract

**Objectives:**

To examine the predictive performance of the Fetal Medicine Foundation (FMF) competing‐risks model for the first‐trimester prediction of a small‐for‐gestational‐age (SGA) neonate in a large, independent, unselected European cohort and to compare the competing‐risks algorithm with previously published logistic‐regression models.

**Methods:**

This was a retrospective, non‐interventional, multicenter cohort study including 35 170 women with a singleton pregnancy who underwent a first‐trimester ultrasound assessment between 11 + 0 and 13 + 6 weeks' gestation. We used the default FMF competing‐risks model for the prediction of SGA combining maternal factors, uterine artery pulsatility index (UtA‐PI), pregnancy‐associated plasma protein‐A (PAPP‐A) and placental growth factor (PlGF) to obtain risks for different cut‐offs of birth‐weight percentile and gestational age at delivery. We examined the predictive performance in terms of discrimination and calibration and compared it with the published data on the model's development population and with published logistic‐regression equations.

**Results:**

At a 10% false‐positive rate, maternal factors and UtA‐PI predicted 42.2% and 51.5% of SGA < 10^th^ percentile delivered < 37 weeks and < 32 weeks, respectively. The respective values for SGA < 3^rd^ percentile were 44.7% and 51.7%. Also at a 10% false‐positive rate, maternal factors, UtA‐PI and PAPP‐A predicted 42.2% and 51.5% of SGA < 10^th^ percentile delivered < 37 weeks and < 32 weeks, respectively. The respective values for SGA < 3^rd^ percentile were 46.2% and 51.7%. At a 10% false‐positive rate, maternal factors, UtA‐PI, PAPP‐A and PlGF predicted 47.6% and 66.7% of SGA < 10^th^ percentile delivered < 37 weeks and < 32 weeks, respectively. The respective values for SGA < 3^rd^ percentile were 50.0% and 69.0%. These data were similar to those reported in the original model's development study and substantially better than those calculated using pre‐existing logistic‐regression models (McNemar's test, *P* < 0.001). The FMF competing‐risks model was well calibrated.

**Conclusions:**

The FMF competing‐risks model for the first‐trimester prediction of SGA is reproducible in an independent, unselected low‐risk cohort and superior to logistic‐regression approaches. © 2025 The Author(s). *Ultrasound in Obstetrics & Gynecology* published by John Wiley & Sons Ltd on behalf of International Society of Ultrasound in Obstetrics and Gynecology.

## INTRODUCTION

Small‐for‐gestational‐age (SGA) fetuses are at higher risk of stillbirth and adverse perinatal outcomes and it is generally accepted that early prediction may reduce the rate of adverse perinatal outcomes related to SGA^1^, ^2^, ^3^, ^4^, ^5^, ^6^, ^7^, ^8^. The Fetal Medicine Foundation (FMF), recognizing the merit of the early prediction and timely identification of SGA, introduced a competing‐risks model for SGA, considering SGA as a spectrum condition to predict during the first, second or third trimester^9^, ^10^, ^11^, ^12^, ^13^, ^14^, ^15^, ^16^, ^17^, ^18^. The severity of the condition is linked to the degree of smallness and the gestational age at delivery and is reflected in the increasing risk according to maternal factors and biomarker levels^9^, ^10^, ^11^, ^12^, ^13^, ^14^, ^15^, ^16^, ^17^, ^18^. This new approach is superior to the traditional methods and widely used cut‐offs, such as pregnancy‐associated plasma protein‐A (PAPP‐A) < 0.4 multiples of the median (MoM), as proposed by the Royal College of Obstetricians and Gynecologists, or fixed biometry cut‐offs, for the prediction and effective stratification of pregnancies at risk for SGA, placental dysfunction‐related stillbirth, adverse neonatal outcome and growth‐related morbidity^17^, ^18^, ^19^, ^20^, ^21^.

External validation in independent populations is an important prerequisite before implementation of a new method of prognosis^22^, ^23^. However, there are limited external validation studies for predicting SGA, primarily focusing on logistic‐regression models, which have demonstrated poor reproducibility^24^. While the FMF competing‐risks model for SGA has undergone extensive internal validation in a series of studies^9^, ^10^, ^11^, ^12^, ^13^, ^14^, and two recent studies have externally validated its performance at mid‐gestation and in the early third trimester^25^, ^26^, only one study, conducted in an Asian population, has shown that the new FMF‐SGA model, when applied in the first trimester, is effective^27^.

The aim of this multicenter study was, first, to externally validate the default FMF competing‐risks model for the first‐trimester prediction of SGA^13^ in a large unselected cohort of women with a singleton pregnancy; and second, to compare the competing‐risks algorithm with previously published logistic‐regression models.

## METHODS

### Study design

This was a retrospective, non‐interventional, multicenter cohort study including unselected women with a singleton pregnancy attending for a routine ultrasound examination at 11 + 0 to 13 + 6 weeks' gestation between January 2012 and October 2023 at seven fetal medicine units across three countries in Europe: Hospital Clínico Universitario Virgen de la Arrixaca, Murcia, Spain; Hospital Universitario de Torrejón, Hospital Universitario Quirón and Hospital Universitario Fundación de Alcorcón, Madrid, Spain; Complejo Hospitalario Universitario A Coruña, Galicia, Spain; Shterev Hospital, Sofia, Bulgaria; and Embriomitriki prenatal diagnostic center, Thessaloniki, Greece.

In this routine assessment, we recorded maternal characteristics and obstetric and medical history, including maternal age, weight, height, race, chronic hypertension, diabetes mellitus Type‐I or ‐II, systemic lupus erythematosus and/or antiphospholipid syndrome, smoking status, mode of conception, parity and information from previous pregnancies, such as birthweight, gestational age at delivery, interpregnancy interval, pre‐eclampsia (PE) or stillbirth. We also performed an ultrasound examination to measure fetal crown–rump length and nuchal translucency thickness, for pregnancy dating and aneuploidy risk assessment, respectively, and to diagnose major fetal defects. Additionally, risk assessment for preterm PE was also performed using a combination of maternal characteristics, mean arterial pressure (MAP), uterine artery pulsatility index (UtA‐PI) and placental growth factor (PlGF), according to the FMF competing‐risks model^28^. The ultrasound scans were performed by sonographers certified by the FMF.

Serum PlGF and PAPP‐A concentrations were measured using one of three automated analyzers: AutoDELFIA or DELFIA Xpress system (PlGF 1‐2‐3 kits; PerkinElmer Inc., Waltham, MA, USA), BRAHMS KRYPTOR analyzer (ThermoFisher Scientific, Hennigsdorf, Germany) or Cobas e411 system (Roche Diagnostics, Mannheim, Germany).

In this study, we included all singleton pregnancies with a live fetus between 11 + 0 and 13 + 6 weeks' gestation that delivered a phenotypically normal neonate at or after 24 + 0 weeks. Pregnancies with chromosomal abnormality and/or major fetal structural abnormality and pregnancies ending in termination, miscarriage or fetal death before 24 weeks were excluded from the study.

The study was approved by the local research ethics committee at each participating center, and the women gave written informed consent to participate.

### Outcome measures

The outcome measures were delivery of a SGA neonate with birth weight below the 10^th^ or 3^rd^ percentile < 37 weeks and < 32 weeks, respectively. The FMF fetal and neonatal population weight charts were used to calculate percentiles and *Z*‐scores for birth weight^29^. Data on pregnancy outcomes were retrieved from hospital/regional records or by contacting the delivering hospitals or the women's general medical practitioners/midwives.

PE was defined according to the American College of Obstetricians and Gynecologists^30^ as chronic or gestational hypertension (systolic blood pressure ≥ 140 mmHg and/or diastolic blood pressure ≥ 90 mmHg, on at least two occasions, 4 h apart) developing after 20 weeks in a previously normotensive woman and at least one of the following: proteinuria (≥ 300 mg/24 h, protein‐to‐creatinine ratio ≥ 30 mg/mmol or urinary dipstick testing ≥ 2+), renal insufficiency with serum creatinine > 97 μmol/L in the absence of underlying renal disease, hepatic dysfunction with blood concentration of transaminases more than twice the upper limit of normal (≥ 65 IU/L for our laboratories), thrombocytopenia (platelet count < 100 000/μL), neurological complications (for example, cerebral or visual symptoms) or pulmonary edema.

### Selection of other models

We searched for published prediction models to validate and directly compare with the competing‐risks approach using specific criteria: first, models were developed and applicable at 11 + 0 to 14 + 0 weeks, including the prediction of preterm SGA; second, multivariate models combining maternal factors with biochemical indices and producing individualized risks; third, availability of the model's equation in the relevant publication; fourth, studies where SGA was the primary outcome and the proposed algorithm explicitly pertained to SGA; and fifth, first‐trimester biomarkers were included.

### Statistical analysis

Descriptive data are expressed as mean ± SD or *n* (%). Initially, we explored the distributional properties of first‐trimester biomarkers, and we also investigated the fit of the FMF birth‐weight charts. UtA‐PI, PAPP‐A and PlGF were converted into MoM, with adjustment for characteristics that were found to provide a substantive contribution to their values^31^. Then, we computed risks using the published default FMF first‐trimester Bayes' theorem‐based competing‐risks model for SGA, including maternal factors, UtA‐PI, PAPP‐A and PlGF^13^. We assessed the discrimination of the model by means of detection rates at a fixed 10% false‐positive rate (FPR) and receiver‐operating‐characteristics curve analysis. Goodness of fit was assessed in terms of calibration intercepts and plots. For perfect calibration (i.e. observed incidence equals the predicted risk of SGA), the slope would be 1.0 and the intercept would be 0. We compared the performance of the model in our validation cohort with the cohort reported in the relevant FMF study^13^ and with the identified published logistic‐regression algorithms arising from the literature search, examining detection rates at a 10% FPR. In particular, McNemar's test was used to compare the detection rates for preterm SGA achieved by the different models at a 10% FPR. The statistical software package R was used for data analysis^32^.

## RESULTS

### Study population

Our cohort included 35 170 women with a singleton pregnancy. The maternal and pregnancy characteristics of the study population are presented in Table 1. Maternal factors, MAP and PAPP‐A data were available for all pregnancies. In 17 862 cases, UtA‐PI was additionally measured and in 9775 cases, PlGF was also measured.

**Table 1:** Maternal and pregnancy characteristics of study population (*n* = 35 170)

Characteristic	Value
Maternal age (years)	32.7 (29.0–36.2)
Maternal weight (kg)	63.5 (57.0–72.5)
Maternal height (cm)	164 (160–168)
Racial origin	
White	34 884 (99.2)
Black	78 (0.2)
South Asian	7 (0.02)
East Asian	19 (0.05)
Asian	9 (0.03)
Mixed	173 (0.5)
Conception	
Natural	31 885 (90.7)
Ovulation induction	375 (1.1)
*In‐vitro* fertilization	2910 (8.3)
Medical history	
Chronic hypertension	389 (1.1)
Diabetes mellitus Type‐I or ‐II	261 (0.7)
SLE/APS	135 (0.4)
Cigarette smoker	4081 (11.6)
Parity	
Nulliparous	18 960 (53.9)
Parous with previous PE	377 (1.1)
Parous with previous SGA*	934 (2.7)
Parous with previous stillbirth	45 (0.1)
Interpregnancy interval (years)	3 (2.0–4.9)
GA at last delivery (weeks)	39.2 (38.6–40.0)
GA at delivery (weeks)	39.5 (38.6–40.4)
Birth weight (g)	3275 (2990–3575)

Data are given as median (interquartile range) or *n* (%).

* Birth weight < 10^th^ percentile.

APS, antiphospholipid syndrome; GA, gestational age; PE, pre‐eclampsia; SGA, small‐for‐gestational age; SLE, systemic lupus erythematosus.

A total of 5092 (14.5%) women delivered a SGA neonate below the 10^th^ percentile, and 2108 (6.0%) delivered a SGA neonate below the 3^rd^ percentile. Overall, 707 (2.0%) women delivered a SGA neonate below the 10^th^ percentile < 37 weeks, and 473 (1.3%) delivered a SGA neonate below the 3^rd^ percentile < 37 weeks. The respective values for delivery < 32 weeks were 0.4% (*n* = 140) and 0.3% (*n* = 108).

Overall, 655 women (1.9%) developed PE. A total of 211 (0.6%) PE cases delivered < 37 weeks and 136 (0.4%) of these pregnancies with preterm PE delivered SGA neonates. There were 444 (1.3%) PE cases that delivered ≥ 37 weeks and 99 (0.3%) of these pregnancies with term PE delivered SGA neonates.

### Application of FMF charts

The incidence of term and preterm SGA was similar to that reported in the FMF study in which the model was developed^13^. The exploratory analysis of the distributional properties of birth‐weight *Z*‐scores demonstrated that the FMF charts had a very good fit to the data (Figure S1). Specifically, the distribution of birth‐weight *Z*‐scores approximated the standard normal distribution. The distribution of birth‐weight *Z*‐scores in relation to gestational age at delivery showed equally spread values around the mean for term pregnancies and a higher incidence of SGA for preterm pregnancies. The latter is the anticipated distributional pattern due to the higher incidence of growth restriction among the preterm pregnancies quantified by the FMF charts.

### Distribution of biochemical indices

The distributions of UtA‐PI, PAPP‐A and PlGF were very similar to published data, both in terms of location and spread (Figure S2), and were similar across all gestational ages.

### Predictive performance of competing‐risks model

**Figure 1:**
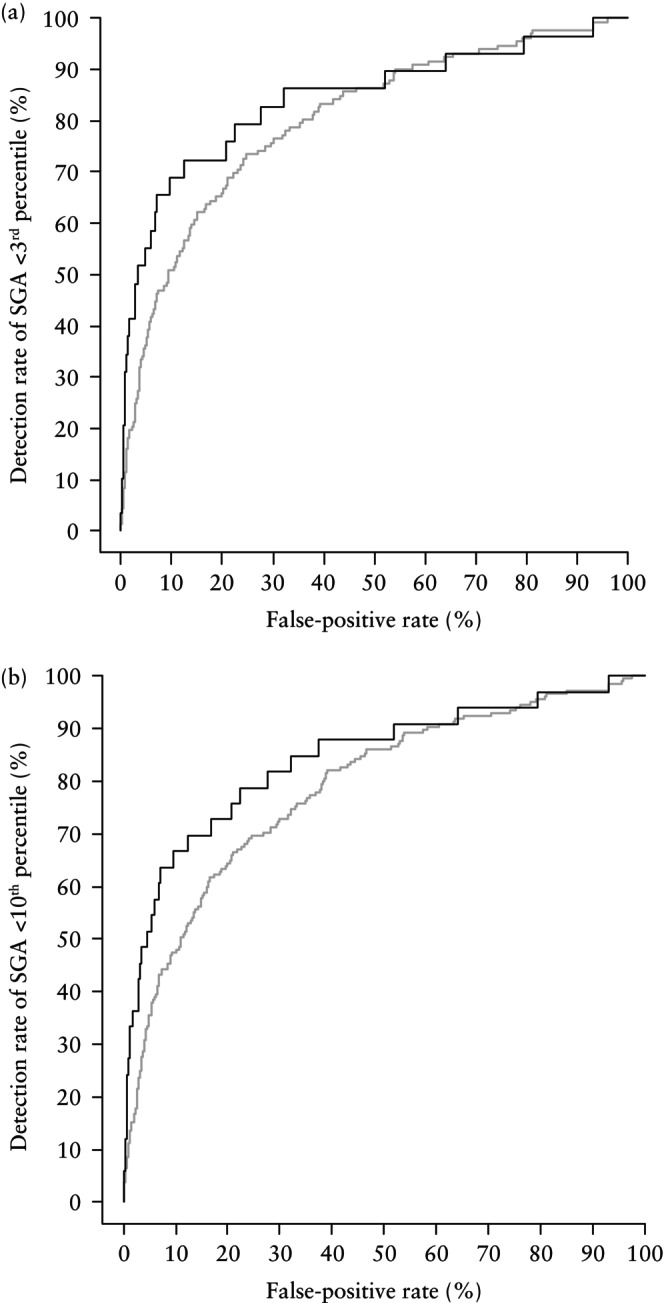
Receiver‐operating‐characteristics curves for prediction of small‐for‐gestational‐age (SGA) neonate < 3^rd^ percentile (a) and < 10^th^ percentile (b) born < 37 weeks (gray) and < 32 weeks' gestation (black) in our validation cohort, using Fetal Medicine Foundation competing‐risks model, which combines maternal factors, uterine artery pulsatility index, pregnancy‐associated plasma protein‐A and placental growth factor.

**Figure 2:**
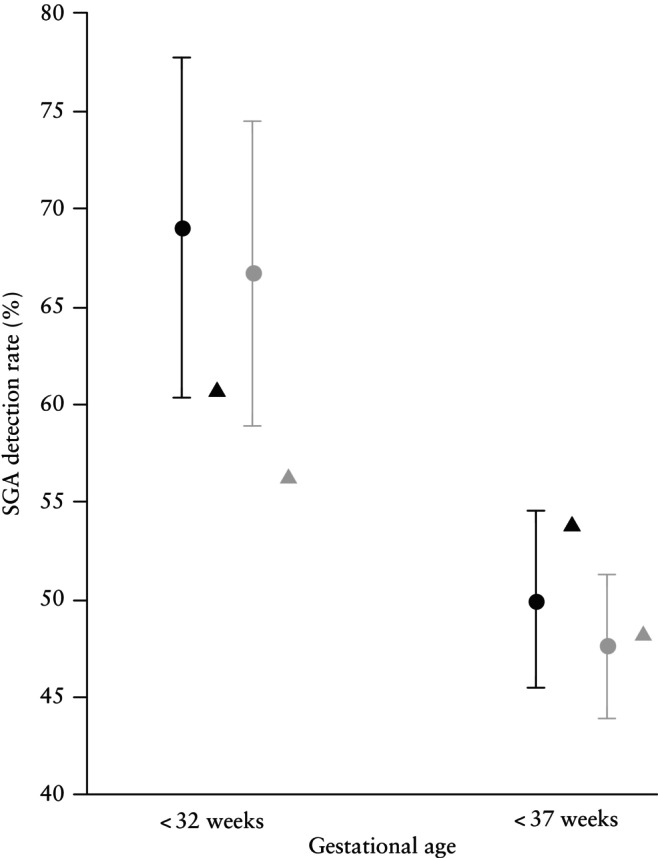
Graph comparing mean detection rates of Fetal Medicine Foundation competing‐risks model for small‐for‐gestational‐age (SGA) neonate < 10^th^ percentile (gray) and < 3^rd^ percentile (black) born < 37 weeks and < 32 weeks' gestation in our validation cohort (circles) and detection rates reported by original FMF study (triangles), which combines maternal factors, uterine artery pulsatility index, pregnancy‐associated plasma protein‐A and placental growth factor, at 10% false‐positive rate. Bars are 95% CI.

**Figure 3:**
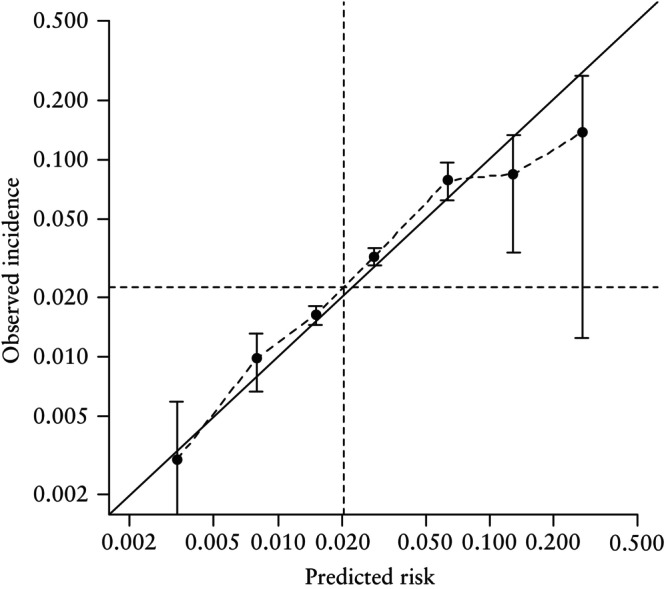
Calibration plot for prediction of small‐for‐gestational‐age neonate < 10^th^ percentile born < 37 weeks' gestation in our validation cohort using Fetal Medicine Foundation competing‐risks model. Horizontal dashed line indicates mean incidence of SGA neonate < 10^th^ percentile born < 37 weeks and vertical dashed line shows mean risk produced by model.

The prediction of a SGA neonate using the combination of maternal factors, UtA‐PI, PAPP‐A and PlGF in the default FMF competing‐risks model is presented in Table 2 and illustrated in Figures 1 and 2. The prediction was better for an increasing degree of prematurity and for SGA < 3^rd^ percentile compared with SGA < 10^th^ percentile, whereas the detection rates were lower for SGA without PE (Figure 1 and Table 2). The detection rates at a 10% FPR in the validation cohort were similar to the published figures of the model's development cohort (Figure 2). PlGF had an incremental value over and above maternal factors and UtA‐PI, improving the detection rate from 42.2% to 49.7% for SGA < 10^th^ percentile born < 37 weeks and from 51.5% to 66.6% for SGA < 10^th^ percentile born < 32 weeks. However, the addition of PAPP‐A did not significantly improve performance. A similar improvement was evident with the addition of PlGF for the prediction of SGA < 3^rd^ percentile (Table 2). The computed risks were in very good agreement with the observed incidence of SGA (Figure 3 and Table 3).

**Table 2:** Performance of prediction by default Fetal Medicine Foundation competing‐risks model of small‐for‐gestational‐age (SGA) neonate in validation cohort

	Outcome measure							
	< 32 weeks' gestation				< 37 weeks' gestation			
	BW < 3^rd^ percentile		BW < 10^th^ percentile		BW < 3^rd^ percentile		BW < 10^th^ percentile	
Screening method	AUC (95% CI)	DR (%) (95% CI)*	AUC (95% CI)	DR (%) (95% CI)*	AUC (95% CI)	DR (%) (95% CI)*	AUC (95% CI)	DR (%) (95% CI)*
MF								
SGA	0.69 (0.60–0.78)	37.9 (28.8–47.1)	0.70 (0.62–0.78)	39.4 (31.3–47.5)	0.72 (0.68–0.76)	34.9 (30.6–39.2)	0.71 (0.68–0.74)	33.0 (29.5–36.5)
SGA without PE	0.69 (0.60–0.78)	33.8 (22.4–45.7)	0.72 (0.65–0.79)	37.4 (27.5–47.8)	0.73 (0.69–0.77)	34.6 (34.2–43.0)	0.72 (0.69–0.75)	35.1 (31.6–38.6)
MF + UtA‐PI								
SGA	0.81 (0.74–0.88)	51.7 (42.3–61.1)	0.80 (0.73–0.87)	51.5 (43.2–59.8)	0.76 (0.72–0.80)	44.7 (40.2–49.2)	0.74 (0.71–0.77)	42.2 (38.6–45.8)
SGA without PE	0.74 (0.66–0.82)	43.8 (34.4–53.2)	0.77 (0.70–0.84)	47.4 (39.1–55.7)	0.71 (0.67–0.75)	38.6 (34.2–43.0)	0.71 (0.68–0.74)	37.3 (33.7–40.9)
MF + UtA‐PI + PAPP‐A								
SGA	0.80 (0.73–0.88)	51.7 (42.3–61.1)	0.79 (0.72–0.86)	51.5 (43.2–59.8)	0.76 (0.72–0.80)	46.2 (41.7–50.7)	0.75 (0.72–0.78)	42.2 (38.6–45.8)
SGA without PE	0.77 (0.69–0.85)	50.0 (40.1–59.4)	0.78 (0.71–0.85)	52.6 (44.3–60.9)	0.74 (0.70–0.78)	43.2 (38.7–47.7)	0.73 (0.70–0.76)	40.3 (36.7–43.9)
MF + UtA‐PI + PlGF								
SGA	0.86 (0.80–0.93)	65.5 (56.5–74.5)	0.86 (0.80–0.92)	66.6 (58.8–74.4)	0.80 (0.76–0.84)	51.5 (47.0–56.0)	0.79 (0.76–0.82)	49.7 (46.0–53.4)
SGA without PE	0.78 (0.70–0.86)	56.3 (47.0–65.7)	0.81 (0.75–0.88)	63.2 (55.2–71.2)	0.75 (0.71–0.79)	44.3 (39.8–48.8)	0.75 (0.72–0.78)	44.0 (40.3–47.7)
MF + PlGF + PAPP‐A								
SGA	0.79 (0.71–0.87)	55.2 (45.8–64.6)	0.79 (0.72–0.86)	54.6 (46.4–62.9)	0.78 (0.74–0.82)	46.2 (41.7–50.7)	0.77 (0.74–0.80)	43.2 (39.6–46.9)
SGA without PE	0.79 (0.71–0.87)	56.3 (47.0–65.7)	0.80 (0.73–0.87)	57.9 (49.7–66.1)	0.78 (0.74–0.82)	45.5 (41.0–50.0)	0.77 (0.74–0.80)	42.5 (38.9–46.1)
MF + UtA‐PI + PAPP‐A + PlGF								
SGA	0.84 (0.77–0.91)	69.0 (60.3–77.7)	0.84 (0.78–0.90)	66.7 (58.9–74.5)	0.80 (0.76–0.84)	50.0 (45.5–54.5)	0.79 (0.76–0.82)	47.6 (43.9–51.3)
SGA without PE	0.79 (0.71–0.87)	62.5 (53.4–71.6)	0.81 (0.75–0.88)	63.2 (55.2–71.2)	0.76 (0.72–0.80)	44.3 (39.8–48.8)	0.76 (0.73–0.79)	43.3 (39.7–47.0)

* At 10% false‐positive rate.

AUC, area under the receiver‐operating‐characteristics curve; BW, birth weight; DR, detection rate; MF, maternal factors; PAPP‐A, pregnancy‐associated plasma protein‐A; PE, pre‐eclampsia; PlGF, placental growth factor; UtA‐PI, uterine artery pulsatility index.

**Table 3:** Calibration study in validation cohort for prediction of small‐for‐gestational‐age neonate with birth weight (BW) < 10^th^ or < 3^rd^ percentile, by maternal factors (MF) and combinations of biomarkers, with delivery < 37 weeks' gestation

	BW < 10^th^ percentile		BW < 3^rd^ percentile	
Screening method	Slope (95% CI)	Intercept (95% CI)	Slope (95% CI)	Intercept (95% CI)
MF	0.98087 (0.8727584 to 1.088982)	−0.11446 (–0.1852343 to –0.0436857)	0.93039 (0.8147521 to 1.046028)	0.07437 (–0.00206859 to 0.150808)
MF + UtA‐PI	0.92334 (0.845157 to 1.0015)	−0.07491 (–0.12207 to –0.02775)	0.86412 (0.7960309 to 0.9322091)	0.13085 (0.05127546 to 0.2104245)
MF + UtA‐PI + PAPP‐A	0.81725 (0.7501016 to 0.8843984)	−0.12003 (–0.1992909 to –0.0407690)	0.75255 (0.6936727 to 0.8114273)	0.06825 (–0.009952 to 0.1464526)
MF + UtA‐PI + PlGF	0.95810 (0.892402 to 1.023798)	−0.10660 (–0.1872917 to –0.02590828)	0.90060 (0.8427811 to 0.9584189)	0.08756 (0.007632669 to 0.1674873)
MF + PlGF + PAPP‐A	0.80671 (0.7478523 to 0.8655677)	−0.23510 (–0.311715 to –0.158485)	0.75160 (0.6982498 to 0.8049502)	−0.09426 (–0.1747557 to –0.0137642)
MF + UtA‐PI + PAPP‐A + PlGF	0.88865 (0.8276951 to 0.9496049)	−0.13216 (–0.2109702 to –0.05334985)	0.82750 (0.7744046 to 0.8805954)	0.05155 (–0.02686816 to 0.1299682)

PAPP‐A, pregnancy‐associated plasma protein‐A; PlGF, placental growth factor; UtA‐PI, uterine artery pulsatility index.

### Comparison with logistic‐regression models

**Figure 4:**
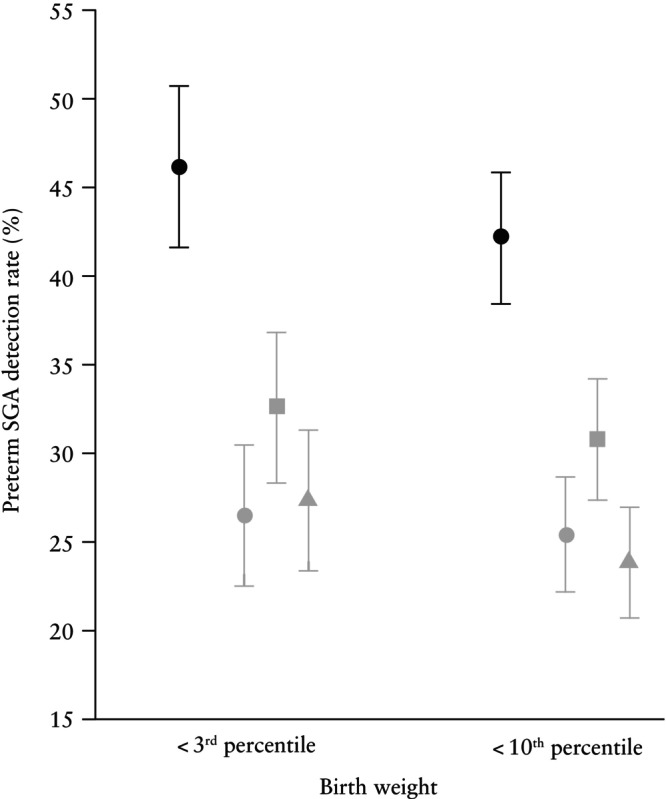
Graph comparing mean detection rates of Fetal Medicine Foundation competing‐risks model (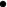) with pre‐existing logistic‐regression models (Crovetto *et al*.^33^ (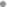), Crovetto *et al*.^34^ (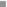) and González‐González *et al*.^35^ (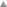)) for preterm small‐for‐gestational‐age (SGA) neonate in validation cohort. Detection was at 10% false‐positive rate and all models included maternal factors, uterine artery pulsatility index and pregnancy‐associated plasma protein‐A, with or without mean arterial pressure. Bars are 95% CI.

We identified three logistic‐regression equations developed in the first trimester explicitly for the prediction of SGA that examined a set of variables including maternal factors, UtA‐PI, PAPP‐A and MAP^33^, ^34^, ^35^. The competing‐risks algorithm used in this study performed substantially better compared with all three pre‐existing models (McNemar's test, *P* < 0.001; Figure 4).

## DISCUSSION

### Main findings

This large, non‐interventional, multicenter cohort study primarily confirms that the FMF competing‐risks model for SGA prediction in the first trimester is reproducible, and demonstrated external validity in a large independent unselected cohort of singleton pregnancies. This study confirms that prediction is better for smaller and more preterm SGA neonates and that the combination of biomarkers and maternal factors improves the prediction provided by maternal factors alone. In particular, higher detection rates are achieved by the model that combines maternal factors, UtA‐PI and PlGF, while the addition of PAPP‐A does not significantly improve performance. Finally, the competing‐risks approach performs substantially better compared with the identified pre‐existing logistic‐regression equations.

### Comparison with previous studies and interpretation of results

The original FMF study assessing the competing‐risks model reported that, at a 10% fixed FPR, the detection rates for a SGA neonate with birth weight < 10^th^ percentile delivered < 37 weeks and < 32 weeks were 43.8% and 48.4%, respectively; and for a SGA neonate with birth weight < 3^rd^ percentile they were 48.7% and 51.0%, respectively^13^. The same figures in our validation cohort were 47.6%, 66.7%, 50.0% and 69.0%, which effectively validates the performance of the original model. The maternal and pregnancy characteristics were similar in our cohort when compared with the UK cohort where the model was originally parameterized, except for the race composition: we included a higher proportion of White women compared with the UK population (99.2% *vs* 73.8%), at the expense of a lower proportion of Black women (0.2% *vs* 17.1%)^13^. Moreover, we examined the distribution of the biomarkers considering several reasons for divergence, such as the quality of the ultrasound marker measurements, laboratory conditions and performance of the MoM conversion equations that could affect the prediction. We found a satisfactory distribution of MoM around 1 for the examined biomarkers and a similar discrimination ability as in the original model. The most recent logistic‐regression algorithm for predicting both SGA and fetal growth restriction (FGR, defined as birth weight < 3^rd^ centile or estimated fetal weight < 10^th^ centile with Doppler abnormalities) at any gestational age in a Spanish population reported detection rates of 42.1% and 66.5%, respectively, at a 10% FPR^34^. Although we believe that the characteristics of this population were similar to those of ours, as about half our cohort was Spanish, we cannot directly compare them since we did not record the Latin–American origin. However, we find indirect comparisons with the results from the different studies unnecessary, as we applied the different algorithms within our same population, enabling direct and equitable comparisons and demonstrating a substantial superiority of the FMF model and its good reproducibility, as previously reported^9^, ^10^, ^13^. The main difference between logistic‐regression models and the FMF Bayesian approach is that the latter considers not only the degree of smallness but also the gestational age at delivery to evaluate the severity of the condition and to estimate the risk. Additionally, the FMF competing‐risks model consists of a flexible algorithm where newer biomarkers can be easily incorporated as they are detected, and particularities of the outcome measure can be adjusted, unlike fixed logistic‐regression models in which adding a predictor or changing the outcome measure requires a different model. This makes logistic‐regression models cumbersome to apply in clinical practice, limiting their use in standardized care pathways and effective population stratification due to poor calibration. The FMF model appears to offer an alternative method that is accurate and reproducible, and provide individual risk estimation for SGA requiring delivery before any specified gestational age and for any degree of smallness.

A key message of this study is that the potential clinical applicability of any new method in diverse settings requires a continuous flexible universal model and a subsequent meticulous validation process based on extensive quality control of the data.

### Clinical and research implications

Before a mathematical model is implemented in clinical practice, its performance should be assessed in different populations from those used to construct the algorithm^22^. However, validating an algorithm in diverse populations is methodologically challenging, and should be based on several principles that are commonly overlooked^22^, ^23^. In this study we have demonstrated the feasibility of expanding the first‐trimester screening program for predicting SGA. After screening, the high‐risk pregnancies could be diverted to a personalized pathway of care that includes interventions such as specialized additional ultrasound scans or blood‐pressure monitoring. Although a second‐trimester assessment is obviously recommended to tailor pregnancy care, this first‐trimester screening may be used in cost‐saving contingency strategies to identify the subgroup of patients that would benefit from a specialized second‐trimester assessment or additional biochemical testing at mid‐gestation^15^. We have also highlighted the importance of extensive quality control of the data, including the biomarker measurements.

It is generally accepted that SGA < 10^th^ or < 3^rd^ percentile for gestational age should be considered a minimum criterion to define FGR, and that some SGA neonates will eventually present with abnormal Doppler results, thereby suffering from growth restriction^8^, ^36^. Early prediction of SGA will allow for timely recognition of growth‐restricted fetuses with signs of hypoxia that will benefit from intense monitoring^18^.

From the research point of view, there is an urgent need to develop prophylactic strategies for SGA, and the best time for any intervention to improve placental function is the first trimester. For instance, a secondary analysis from the ASPRE and SPREE trials demonstrated that administering aspirin (150 mg/day) to women at high‐risk for PE, starting from 11 to 14 weeks' gestation, reduces the risk of SGA < 10^th^ percentile by about 40% in those born < 37 weeks and by about 70% in those born < 32 weeks^37^. This external validation of the FMF competing‐risks model for the prediction of SGA may serve as a foundation for selecting the high‐risk population to be included in a randomized controlled trial for this purpose.

### Strengths and limitations

To the best of our knowledge, this is the largest external validation study for SGA prediction conducted in the first trimester. We adhered to the methodological principles of conducting and reporting an appropriate validation study^22^, ^23^, ^24^. We followed the recommended validation process, adhering to important aspects that may affect the accuracy of prediction. For instance, we ensured the satisfactory fit of the FMF birth‐weight charts, indicating their feasibility for clinical use and that the observed incidence of SGA was the anticipated one. The model specifications were explicitly defined in the cited studies, and full parameterization without rounded coefficients was available. Moreover, this large, consecutive, multicenter, European validation cohort provided reliable information about the incidence of the examined outcome and the frequency distributions of the risk factors and biomarkers used, and maintained consistency with the outcome definition used in the original studies. Finally, we directly compared the performance of each model by applying them within the same population, facilitating robust evaluation.

A limitation of this study may be its retrospective nature. However, the data were prospectively and consecutively collected from an unselected population in the context of everyday clinical practice. We did not perform a proper health economic analysis, but using this model does not require extra resources, and cost savings are plausible owing to the possible reduction of adverse outcomes related to SGA.

### Conclusions

This study has demonstrated that the competing‐risks model for SGA is reproducible, with a performance similar to that reported in the original studies^9^, ^10^, ^11^, ^13^. It appears that this approach is a reliable tool that could easily be adapted into routine prenatal care, utilizing the pre‐existing infrastructure. Future trials should investigate whether prediction, prevention strategies and/or stratification of care are beneficial for improving perinatal outcomes.

## Supporting information


**Figure S1** (a) Distribution of birth‐weight percentiles and relationship with gestational age at delivery, using Fetal Medicine Foundation (FMF) charts in our validation cohort. Dashed lines show different percentiles. (b) Distribution of birth‐weight *Z*‐scores after adjustment for gestational age using FMF charts. Birth‐weight percentiles are depicted for simplicity of interpretation.
**Figure S2** Box‐and‐whiskers plots showing distribution of biomarkers used in prediction of small‐for‐gestational‐age neonate in our validation cohort. Boxes show median and interquartile range, and whiskers are range. MOM, multiples of the median; PAPP‐A, pregnancy‐associated plasma protein‐A; PlGF, placental growth factor; UtA‐PI, uterine artery pulsatility index.

## Data Availability

The data that support the findings of this study are available on request from the corresponding author. The data are not publicly available due to privacy or ethical restrictions.
