# Supplementary figures and images for: External validation and comparison of Fetal Medicine Foundation competing‐risks model for small‐for‐gestational‐age neonate in the first trimester: multicenter cohort study
Source: Ultrasound Obstet Gynecol. 2025 Apr 14;65(6):729–37. doi: 10.1002/uog.29219 (PMC12127715; doi:10.1002/uog.29219)

## Slide 1
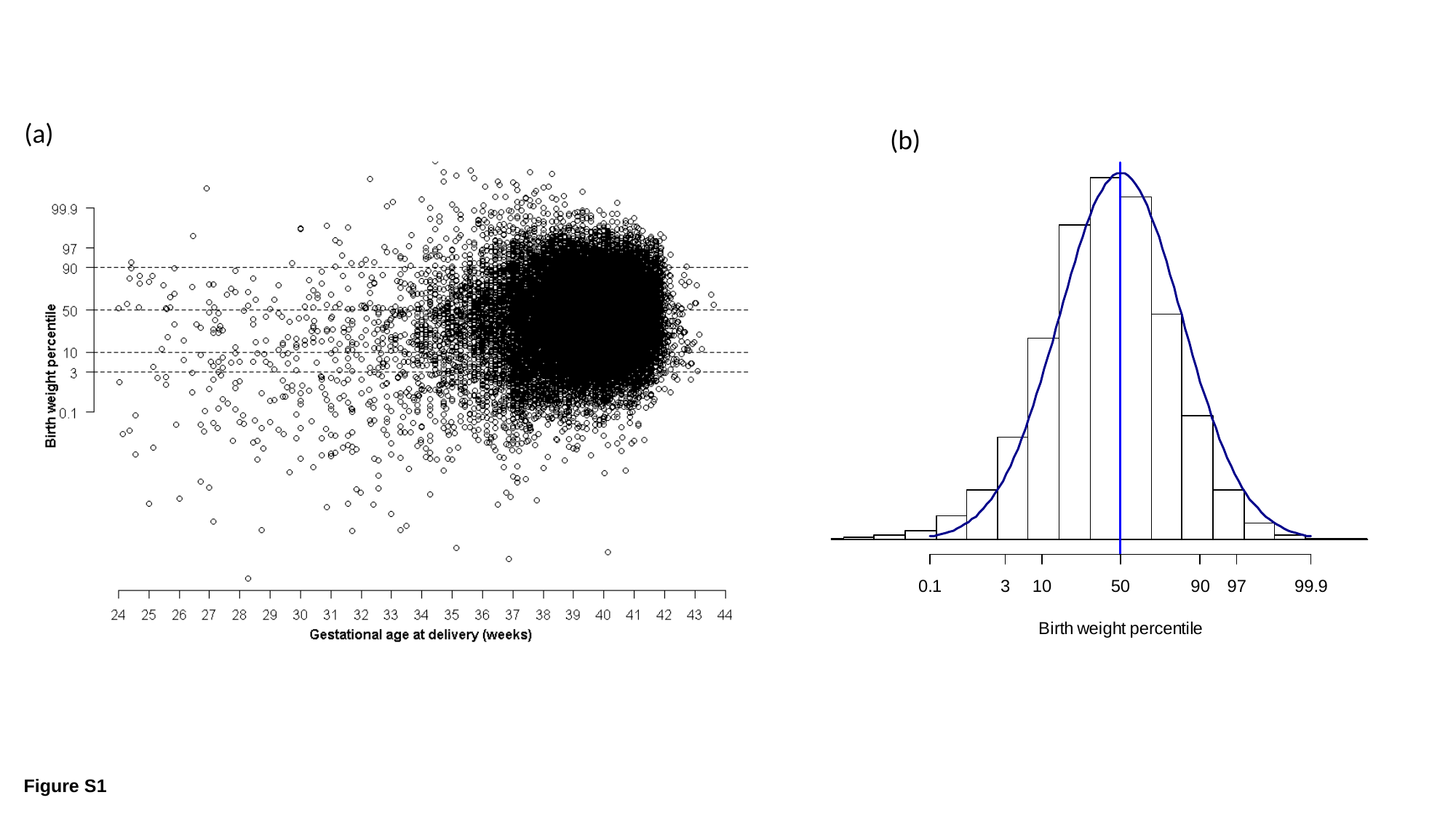

(a)
(b)
Figure S1

## Slide 2
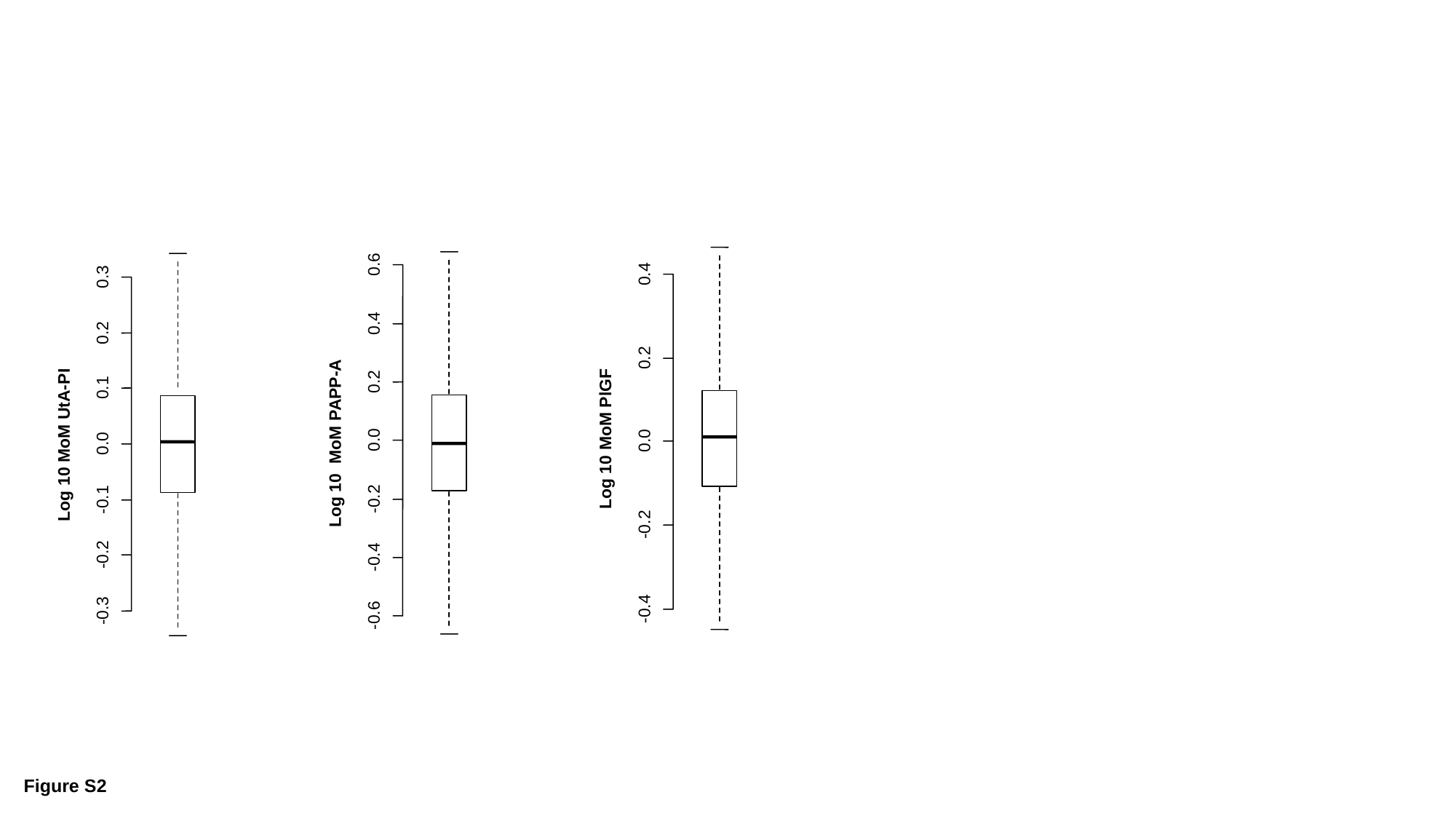

0.4
0.2
0.0
Log 10 MoM PlGF
-0.2
-0.4
0.6
0.4
0.2
0.0
Log 10 MoM PAPP-A
-0.2
-0.4
-0.6
0.3
0.2
0.1
0.0
Log 10 MoM UtA-PI
-0.1
-0.2
-0.3
Figure S2

Supplement: Supplementary file 1 — Figure S1 (a) Distribution of birth‐weight percentiles and relationship with gestational age at delivery, using Fetal Medicine Foundation (FMF) charts in our validation cohort. Dashed lines show different percentiles. (b) Distribution of birth‐weight Z‐scores after adjustment for gestational age using FMF charts. Birth‐weight percentiles are depicted for simplicity of interpretation. Figure S2 Box‐and‐whiskers plots showing distribution of biomarkers used in prediction of small‐for‐gestational‐age neonate in our validation cohort. Boxes show median and interquartile range, and whiskers are range. MOM, multiples of the median; PAPP‐A, pregnancy‐associated plasma protein‐A; PlGF, placental growth factor; UtA‐PI, uterine artery pulsatility index. [file UOG-65-729-s001.pptx]
